# Changes in Brain Metallome/Metabolome Pattern due to a Single *i*.*v*. Injection of Manganese in Rats

**DOI:** 10.1371/journal.pone.0138270

**Published:** 2015-09-18

**Authors:** Katharina Neth, Marianna Lucio, Alesia Walker, Julia Zorn, Philippe Schmitt-Kopplin, Bernhard Michalke

**Affiliations:** 1 Research Unit Analytical BioGeoChemistry, Helmholtz Zentrum München—German Research Center for Environment and Health (GmbH), Ingolstädter Landstrasse 1, D-85764, Neuherberg, Germany; 2 Research Unit Comparative Medicine, Helmholtz Zentrum München—German Research Center for Environment and Health (GmbH), Ingolstädter Landstrasse 1, D-85764, Neuherberg, Germany; 3 Chair of Analytical Food Chemistry, Technische Universität München, Alte Akademie 10, D- 85354, Freising-Weihenstephan, Germany; CINVESTAV-IPN, MEXICO

## Abstract

Exposure to high concentrations of Manganese (Mn) is known to potentially induce an accumulation in the brain, leading to a Parkinson related disease, called manganism. Versatile mechanisms of Mn-induced brain injury are discussed, with inactivation of mitochondrial defense against oxidative stress being a major one. So far, studies indicate that the main Mn-species entering the brain are low molecular mass (LMM) compounds such as Mn-citrate. Applying a single low dose MnCl_2_ injection in rats, we observed alterations in Mn-species pattern within the brain by analysis of aqueous brain extracts by size-exclusion chromatography—inductively coupled plasma mass spectrometry (SEC-ICP-MS). Additionally, electrospray ionization—ion cyclotron resonance-Fourier transform-mass spectrometry (ESI-ICR/FT-MS) measurement of methanolic brain extracts revealed a comprehensive analysis of changes in brain metabolisms after the single MnCl_2_ injection. Major alterations were observed for amino acid, fatty acid, glutathione, glucose and purine/pyrimidine metabolism. The power of this metabolomic approach is the broad and detailed overview of affected brain metabolisms. We also correlated results from the metallomic investigations (Mn concentrations and Mn-species in brain) with the findings from metabolomics. This strategy might help to unravel the role of different Mn-species during Mn-induced alterations in brain metabolism.

## Introduction

Manganese (Mn) is an essential trace element needed for proper functionality of various physiological processes, but can also be very harmful to the body. When Mn is taken up in higher concentrations than recommended, it is transported into the brain via the bloodstream, where it can accumulate in the region of basal ganglia affecting dopaminergic nigrostriatal metabolisms [1]. This results in a disease called manganism showing similarities to Parkinson´s Disease (PD) due to the overlap in affected brain areas [2]. Moreover, the role of Mn in the development of PD has also been discussed [3].

The major hazardous route of Mn-uptake is inhalation during occupational exposure under insufficient worker protection [4–6]. However, environmentally inhalable concentrations of Mn are also increasing due to the use of an organic, Mn-containing anti-knock agent in gasoline or Mn-containing pesticides in some countries [7–11]. Also inhabitants of regions nearby ferroalloy producing industry were shown to develop predominantly parkinsonian disturbances due to the excess of inhaled Mn concentrations over prolonged time [12].

However, the mechanisms underlying the resulting neuronal inflammation and degradation seem to consist of multifactorial processes. Excitatory neurotransmitters such as glutamate or inhibitory neurotransmitters such as GABA were shown to be affected mediators in Mn-induced neuroinflammation [13, 14]. Antioxidative enzymes such as Mn-SOD (Mn superoxide dismutase) or acetylcholinesterase also seem to lose their protective functions against oxidative stress during Mn overexposure, assuming that mitochondria play a major role in the mechanism of Mn-induced toxicity [15]. Even on molecular level some studies have shown that Mn treatment of cells resulted in a decrease of energy related nucleotides failing in regulation of cellular energy and redox state [16]. Therefore, Mn-induced neural pathophysiological processes seem to be versatile and may affect several metabolic pathways.

Studies mostly describe the application of different Mn compounds *in vitro* or *in vivo* and can therefore only observe effects of applied total Mn. The certain Mn-species, which might be crucial for crossing neural barriers, carrying Mn into the brain and therefore affecting brain metabolisms, remains unknown. So far, few studies could show that predominantly low molecular mass (LMM) compounds, like citrate, are able to bind and transport Mn across neural barriers at a certain exposure or concentration of Mn [17–19]. In a previous study, Diederich et al. applied a single low dose Mn injection (1 mg/kg b.w., i.v.), which revealed that, besides inorganic Mn, Mn-citrate was the predominant Mn-species in rat brain extracts of exposed animals [20].

Based on that study, we herein applied a similar concentration of MnCl_2_ (1.5 mg/kg b.w., i.v.) for a single time in rats with the aim to examine metabolite changes in brain. One major aim of the application of this low concentration was to achieve levels of Mn within the animal, which are comparable to concentrations of Mn in humans after a single contact (e.g. accident at work or injection of Mn-containing contrast agents for MRI [21, 22]. To reveal alterations in neural metabolisms, metabolite analysis was carried out in methanolic rat brain extracts by electrospray ionization—Fourier transform—ion cyclotron resonance—mass spectrometry (ESI-ICR/FT-MS). With the power of this metabolomics analysis, the versatile effects of Mn on different metabolic pathways in brain could be observed in a non-targeted manner. This might allow the detection and the linkage of different metabolic interactions resulting from a harmful Mn-exposure as applied herein. On the other hand, we also studied Mn-speciation in brain by hyphenation of a separation technique to a sensitive elemental detector ICP-MS (inductively coupled plasma mass spectrometer), referring to the previous study by Diederich et al. Since Mn-species naturally are very labile, size exclusion chromatography (SEC) of aqueous brain extracts was chosen as separation technique. The additional combination of metabolomics with the metallomic approach allowed us to enter a completely new field for investigating of Mn-induced neural alterations. Our study could be a first step towards unraveling important Mn-species, which are more harmful than others, and might therefore lead to a more detailed understanding of the ongoing transport procedure or mechanisms of action after Mn exposure.

## Results and Discussion

### Mn concentrations in brain, brain extracts or pellets and feces

Following a single i.v. injection of either NaCl in control rats or 1.5 mg Mn/kg b.w. in test rats (+Mn), feces were collected for four days until sacrifice of animals. As biliary excretion is the main route of Mn excretion [23], analysis of feces was chosen to monitor excretion after injection (see Text A in S1 File). Interestingly, Mn concentration in feces decreased over the four days of measurement with excretion values on day four comparable to the ones of control rats (Fig A in S1 File). This means that excretion of Mn in Mn-exposed rats was balanced soon after injection and also reflects the strict control of Mn-excretion via the bile to feces.

Brains were taken for the determination of Mn concentrations and for an aqueous brain extraction applied for Mn-speciation. Table 1 summarizes measured Mn concentrations in total brain, aqueous brain extracts and pellets, which were obtained during the extraction procedure.

**Table 1:** Concentrations of Mn in brain extracts, pellets, and total brain as well as extraction efficiency (EE).

	Control	+Mn
**Extract (ng/g)**	108.6 ± 4.8	165.1 ± 18.0***
**Pellet (ng/g)**	262.4 ± 9.1	285.1 ± 20.9*
**Total (ng/g)**	360.1 ± 18.0	429.1 ± 15.9***
**Extract+Pellet (%)**	113.6 ± 11.8	104.7 ± 5.2
**EE(%)**	34.1 ± 3.7	38.4 ± 2.7

Total Mn concentration in brain was elevated (+19%) due to the injection of MnCl_2_ compared to control. Extraction efficiency was high enough to use the extracts for Mn-speciation.

*p<0.05,

***p<0.001.

Due to the single injection of MnCl_2_, Mn concentrations in the brain were significantly elevated by 19% compared to control rats in total brain (429.1 ng/g and 360.1 ng/g, respectively, p<0.001). This observation also shows that despite of declining Mn excretion via feces in Mn exposed rats, accumulation in the brain occurred. Extraction of brain tissue led to significantly elevated levels of Mn in extracts and pellets of treated rats with an averaged extraction efficiency of 36.3±3.2%. This ensured a highly efficient extraction procedure compared with the results from the developed extraction method [24]. Therefore, brain extracts could be used for analysis of Mn-species distribution in brain by means of SEC-ICP-MS.

### Mn-speciation in brain by SEC-ICP-MS

**Fig 1:**
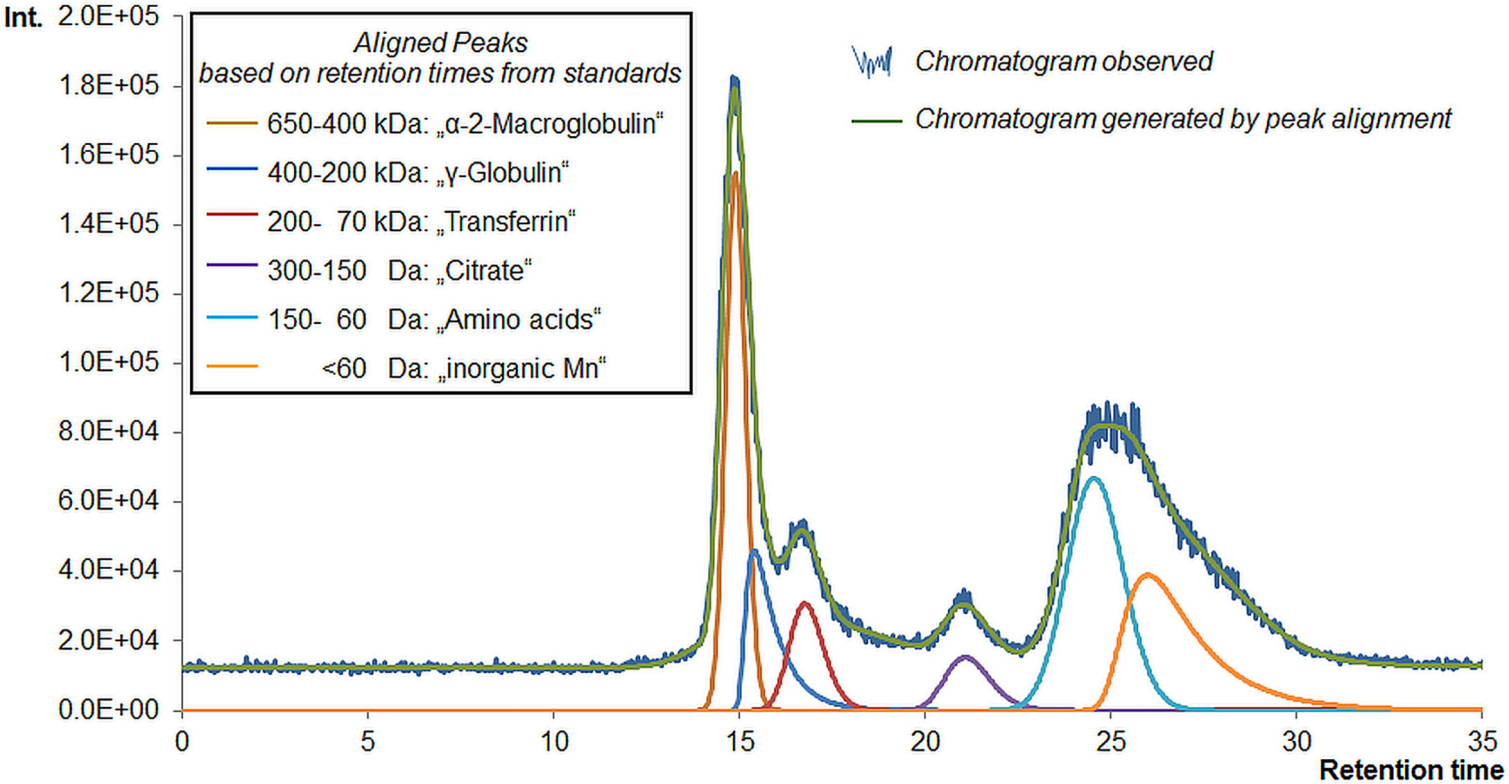
SEC chromatogram of a brain extract with peak alignment. The figure shows exemplarily a SEC chromatogram of one brain extract. SEC fractions were determined according to retention times of respective Mn standards as shown by colored peaks. The final peak alignment and area calculations were carried out by application of PeakFit^TM^ software (green line, generated/aligned chromatogram).

Hyphenation of size exclusion chromatography (SEC) to ICP-MS was performed to determine the distribution of Mn amongst different carrier molecules in the aqueous brain extracts of Mn injected and control rats. Fig 1 demonstrates exemplarily a native chromatogram of a brain extract and the corresponding peak alignment by PeakFit^TM^ software.

**Fig 2:**
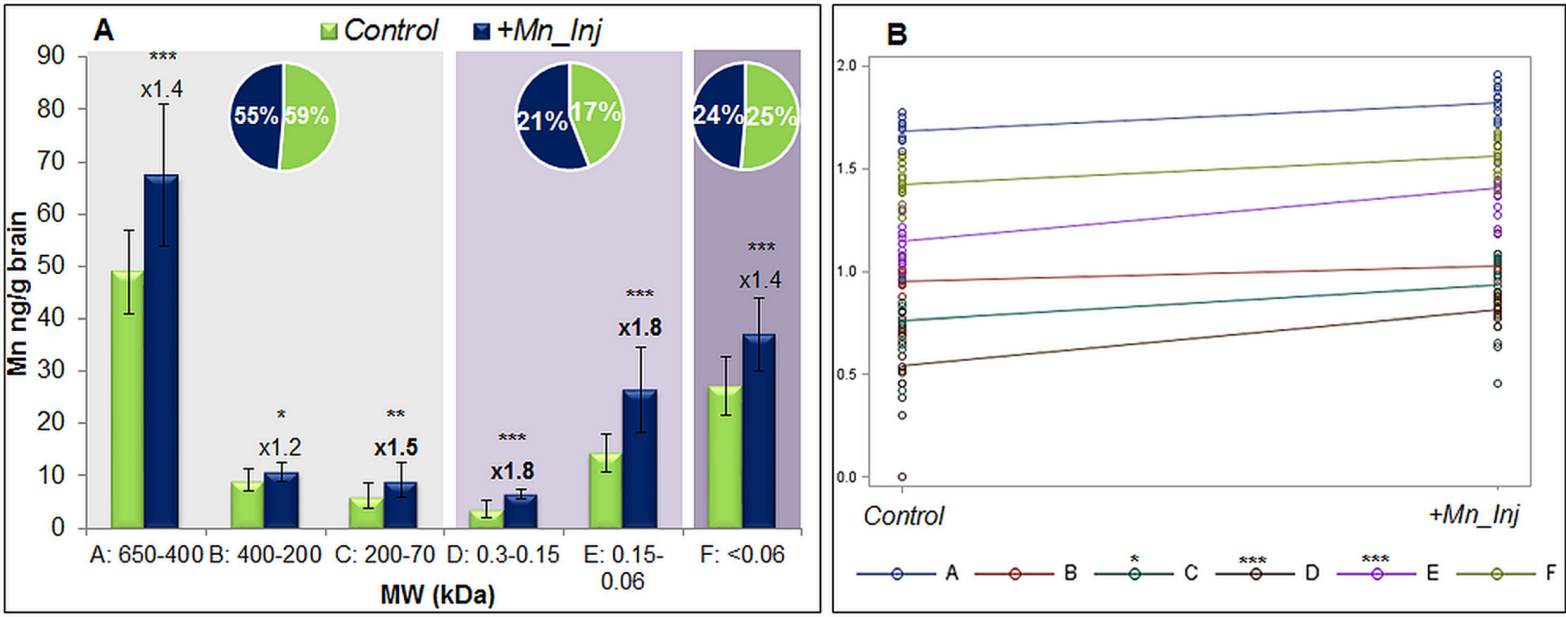
Concentrations of Mn from different species in brain extracts according to SEC-ICP-MS. (A) The figure shows by columns the respective Mn concentrations of SEC fractions A-F (MW in kDa) for control and +Mn samples as well at the fold-change in increase (n = 15 per group). The pie charts show the percentage of Mn in each group (HMM, LMM or inorganic Mn) compared to total Mn in the aqueous brain extracts. A slight decrease in brain HMM was accompanied by an increase of Mn at LMM carriers in Mn-exposed animals. (B) Interaction plot from two-way ANOVA of results from Mn-speciation. Fractions C, D, and E were shown to have significant effects compared to the control group, which is in line with the findings in the fold-changes from t-test. *p<0.05, **p<0.01, ***p<0.001.

Based on retention times determined by analysis of prepared Mn-standards, six different size fractions were separated: 650–400 kDa (“α-2-Macroglobulin”, fraction A), 400–200 kDa (“γ-Globulin”, fraction B), 200–70 kDa (“Transferrin”, (Tf), fraction C), 0.3–0.15 kDa (“Citrate”, (Cit), fraction D), 0.15–0.06 kDa (“Amino acids”, fraction E) and <0.06 kDa (“inorganic Mn”, fraction F). Quotation marks are used to show that the applied standards were prepared as possible binding partners of Mn according to obtained retention times and literature comparison [25]. Of course, other unknown carrier molecules might also be eluting in the same time frame. SEC only allows setting of eluting fractions due to its limited resolution, but is still the most suitable separation method for the inherently very labile Mn-species. In this study it was important to distinguish between high molecular mass compounds (HMM) and low molecular mass compounds (LMM), especially Mn-citrate. These were separated to our convenience by the applied method, and a further LMM fraction as well as inorganic Mn could accordingly be separated. The Mn concentrations found for the different species in brain extracts of control and Mn-exposed rats calculated from peak areas are shown in Fig 2.

Compared to control samples, Mn concentrations were significantly increased in all fractions of Mn-injected rat brain extracts (+Mn) as shown in Fig 2A. The strongest increase was observed for fractions D and E (both 1.8-fold, p<0.001), followed by fraction C (1.5-fold, p<0.01) and fractions A and F (both 1.4-fold, p<0.001). Regarding fraction B, no remarkable difference was observed between control and +Mn_Inj samples (1.2-fold increase, p<0.05). The significance of differences between control and +Mn_Inj samples was also analyzed by a two-way ANOVA including the interaction effect between group (i.e. control or Mn-exposed) and fraction (i.e. A-F), which is shown in the interaction plot in Fig 2B. Such analysis is more valid compared to a t-test as it includes more than just one factor. Hence, the most important effects for Mn-species formation came from fraction C (Tf, p = 0.0165) and the LMM fractions D (citrate) and E (amino acids, both p<0.001). The respective p-values can be found in Table A in S2 File. The results are therefore substantiating the observations of the fold changes, where fractions C, D and E showed highest increases due to Mn-exposure. Interestingly, in serum, we observed major effects for Mn-species formation for fractions C and E after this short exposure [26]. For transportation across neural barriers, also Mn-citrate (fraction D) seemed to be important, which is in line with previous speciation studies, where an active transport of LMM Mn-species across neural barriers was hypothesized [19, 20, 27, 28]. So far it is assumed that preliminary Mn-citrate is formed during Mn exposure at certain concentrations. This formation of Mn-citrate might already occur in serum and be responsible for transportation of Mn into the brain. The importance of citrate as Mn carrier was already stated by Yokel and Crossgrove, who found a three-fold higher influx co-efficient from blood to brain for Mn-citrate than for inorganic Mn or Mn-Tf [17]. Also Michalke et al. could observe the increased formation of Mn-citrate in paired human serum/cerebrospinal fluid (CSF) samples, where the authors also suggested to make use of Mn-citrate concentrations in serum for Mn biomonitoring [18]. The herein observed increase in fraction E (amino acids) due to Mn injection supports earlier studies where the formation of several Mn-amino acid complexes was observed in human CSF by CZE (capillary zone electrophoresis)-ICP-MS [29]. The pie charts in Fig 2A depict the percentage sum of HMM, LMM and inorganic Mn resulting from SEC-fractions compared to total Mn in the aqueous brain extracts. Although changes are not very high, a reduction in HMM compounds accompanied by an increase in LMM Mn-species could be observed for +Mn_Inj samples. These only slight changes could be due to the applied low concentration of Mn. The comparable high amount of HMM species in brain extracts was not expected by us. However, we assume this to be related with artefacts from samples as at the same time of analysis, brain extracts from another Mn-exposure study were analyzed in randomized order. There, the majority of Mn was predominant as LMM Mn-species or inorganic Mn (unpublished data); hence, an analytical problem could be excluded. As the high amount of HMM herein was also fact for control samples, we assume that the animals inherently possessed more HMM Mn-species or species transformation during sample preparation or storage might have occurred. Such shift towards HMM compounds was also observed in other studies of Mn-speciation by SEC-ICP-MS (personal communication). Nevertheless, although comparable low amounts of Mn were applied in this study, a shift from HMM towards LMM compounds for Mn transportation in brain could be observed as discussed above.

### Changes in brain metabolisms determined by means of ESI-ICR/FT-MS

**Fig 3:**
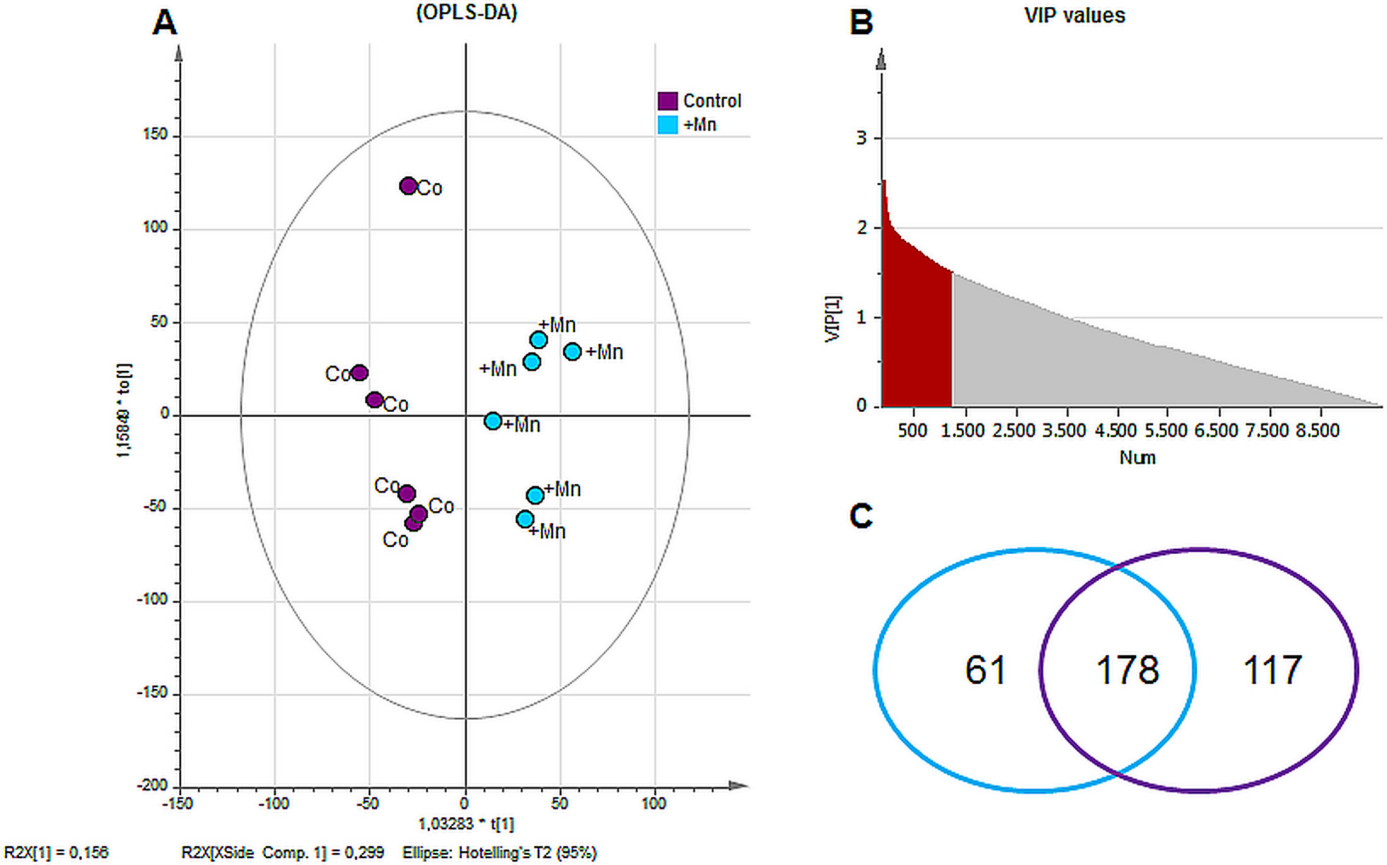
Data elaboration from ESI-FT-ICR-MS measurement. (A) Score Scatter Plot of OPLS-DA of detected metabolites by ESI-ICR/FT-MS revealed a good separation between control (Co) and Mn exposed group (+Mn). (B) Distribution of VIP values (variables important in projection) of metabolites from OPLS-DA: metabolites with values ≥1.50 were chosen to be important for group separation (important metabolites, in grey). (C) Annotation of detected masses by MassTRIX webserver revealed in total 1332 known masses. According to multivariate analysis, 178 of the annotated/known metabolites were determined as important metabolites with 117 metabolites being characteristic for the control group and 61 metabolites being characteristic for +Mn group.

A non-targeted metabolomics study was performed in order to analyze effects on brain metabolism of a single i.v. Mn injection in rats. An ESI-ICR/FT-MS analysis was carried out in methanolic brain extracts. In sum, 9865 masses were detected in all extracts, where 1332 masses (≈13.5%) have been annotated by MassTRIX webserver [30] and they are therefore determined as “known” metabolites. OPLS-DA of the entire dataset revealed a good separation between the control and Mn-treated group (score scatter plot in Fig 3A). The model gave good values for R^2^Y(cum) = 0.8 and Q^2^(cum) = 0.5 (indices representing the goodness of the fit and the prediction ability of the model, respectively). Annotated/known metabolites comprising a VIP value (variables of importance in projection) greater than 1.5 (Fig 3B) were considered as “important metabolites”, summarizing the overall contribution of each X-variable and weighted according to the Y (in this case the two different classes) variation accounted for by each component. Among these important metabolites (178 in total), 117 were assigned to the control and 61 to the +Mn group as shown in the Venn diagram in Fig 3C.

**Fig 4:**
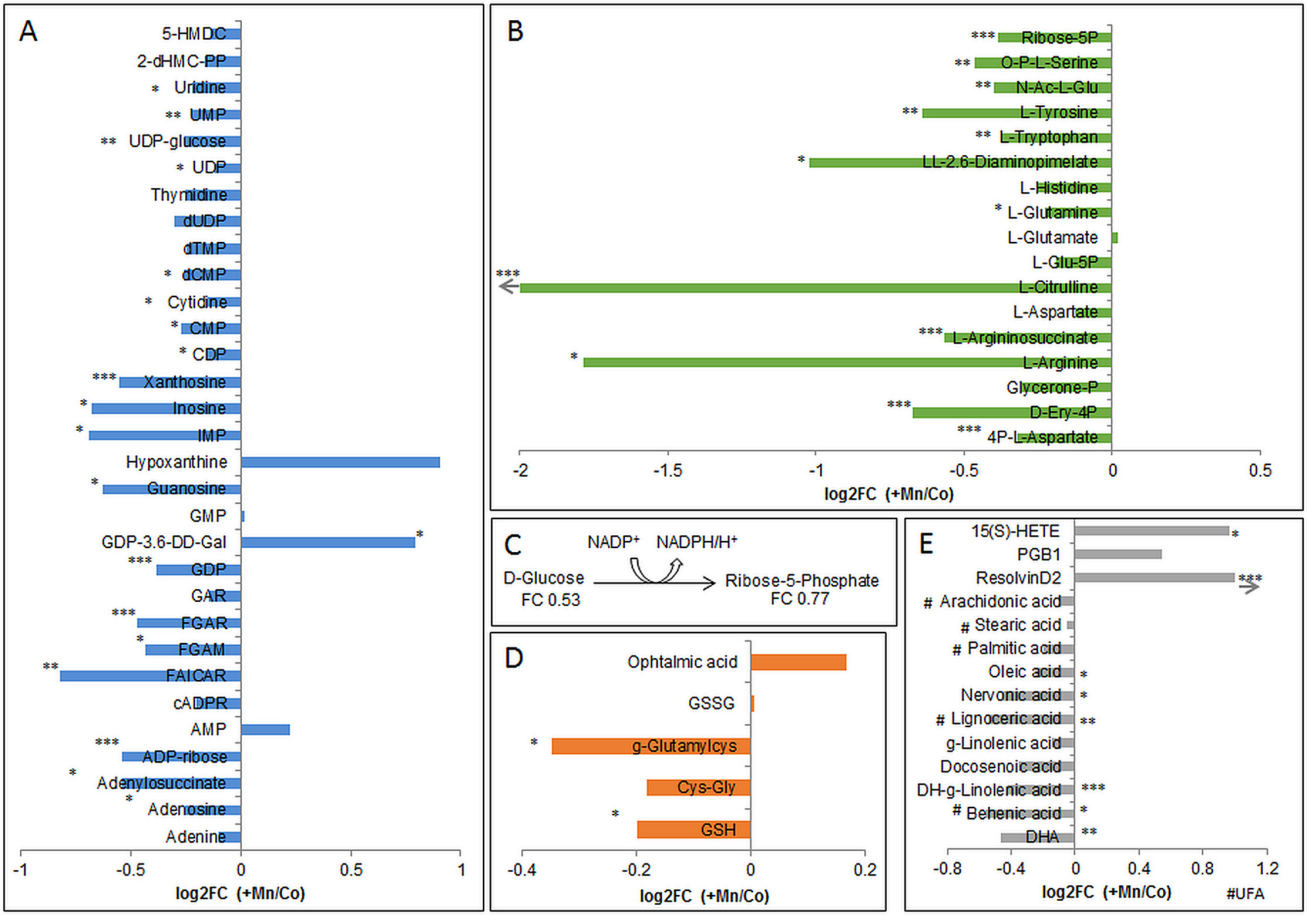
Changes in major brain metabolisms. (A) Alterations in metabolites of purine and pyrimidine metabolism. (B) Alterations in amino acid metabolism. (C) Reaction of glucose to ribose-5-phosphate for energy production with fold changes (+Mn/Co). (D) Changes in glutathione metabolism (GSH = glutathione, GSSG = glutathione disulfide, Cys-Gly = cysteinylglycine). (E) Changes in synthesis of fatty acids (15(S)-HETE = 15(S)-Hydroxyeicosatetraenoic acid, DHA = Docosahexaenoic acid, PGB1 = prostaglandinB1, g = gamma, DH = dihomo; UFA = unsaturated fatty acid). *p<0.05, **p<0.01, ***p<0.001 t-test between intensities of control and +Mn samples; arrow means that value exceeded the maximum of axis.

Annotated metabolites were further analyzed by KEGG color pathway to find changes in involved metabolic pathways. Major changes could be observed for purine and pyrimidine Fig 4A), amino acid (Fig 4B), glucose (Fig 4C), and glutathione metabolism (Fig 4D) as well as for biosynthesis of fatty acids (Fig 4E).

#### Purine and pyrimidine metabolism

Interestingly, almost all detected purines or pyrimidines were significantly decreased in +Mn rat brain extracts except for hypoxanthine, AMP (adenosine monophosphate), GMP (guanosine monophosphate) and GDP (guanosine diphosphate)-3,6-dideoxy-galactose, which were slightly increased. Purine nucleosides are involved in a variety of intracellular neuronal processes and provide precursors of RNA and DNA [31]. Over all, adenosine, guanosine, inosine and uridine participate in protection of versatile neurological insults, e.g. in pathophysiological processes during PD [32]. Inosine was also shown to be neuroprotective by its breakdown product ribose-1-phosphate, to inhibit poly-(ADP-ribose) polymerase and to interact with adenosine receptors [31]. With inosine being the breakdown product of adenosine, the effects of increase or decrease in their concentration are mediated by interaction with adenosine receptors (A_1_, A_2A_, A_2B_, A_3_). Interestingly, A_2A_-receptors form heteromeric complexes with dopamine- and A_1_-receptors enabling purines to fine-tune neurotransmission in basal ganglia [33, 34]. This interaction also includes an inverse regulation of glutamate, serotonin and acetylcholine excretion via activation of A_1_ receptors, what further elevates neuroprotective mechanisms [33, 35–37]. In short, this means the more purines interact with adenosine receptors, the less of mentioned neurotransmitters are excreted. A decrease of adenosine after chronic Mn treatment was also observed by Villalobos et al. [38], especially in brain areas, which are known to be more susceptible for Mn toxicity. Furthermore, adenosine possesses anti-inflammatory effects as it is able to reduce the toxic effects of oxygen radicals [35]. The reduction of adenosine levels might prompt the formation of free radicals alleviating Mn-induced neuroinflammation. Moreover, a negative correlation of guanosine in blood with Mn concentrations in brain was already described by Dorman et al. [39] after a chronic Mn inhalation in rhesus monkeys. Hence, guanosine is also able to promote glutamate uptake and reduce oxidative stress [40]. In summary, the observed reduction in biochemical important purines like adenosine, guanosine or inosine in rat brain due to a single low dose Mn injection might lead to an inactivation of these neuroprotective effects.

#### Amino acid metabolism

The disturbed purine metabolism is connected to a disturbed amino acid metabolism by the interaction of adenosine with dopamine receptors and therefore also affecting the excitatory excretion of neurotransmitters. We observed a decrease in all detected amino acids, except for glutamate, which implied an increase due to Mn-exposure in rat brain (Fig 4B). Special focus in context with Mn-exposure has to be given on enzymes of amino acid synthesis like tyrosine hydroxylase (TH) or glutamine synthetase (GS). With TH being the rate limiting enzyme in dopamine synthesis, it produces dopamine from tyrosine by the intermediate L-Dopa. A reduction in brain dopamine levels due to increasing Mn concentrations has been described in different studies [41–44]. However, activation of TH seems to be dependent on duration of Mn exposure which is consistent with Zhang et al. [45]. An inactive TH might also be the result of a deficit of the precursor amino acid L-Tyrosine as observed herein. With Mn being a cofactor of GS [46], it is involved in formation of glutamine from glutamate by addition of ammonia. The interference of Mn with this enzyme was also studied by Deng et al. who observed an inhibition of GS in astrocytes in a concentration dependent manner [47]. As discussed in [48] GS protein levels were also altered in airborne Mn-exposed monkeys [49] and sensitivity of GS might come from the Mn-induced oxidative stress. Overall, the observed decrease in brain L-Glutamine levels in Mn-injected rats militates for an inactivation of GS leading to failure in glutamine synthesis. However, an increase in L-Glutamate was only indicative in Mn-treated rat brains, but the increase or decrease of glutamate levels seems again to be dependent on Mn concentration [50, 51]. We further detected nine out of the 20 proteogenic amino acids, which were decreased in rat brain tissue due to Mn injection. This might account for a perturbed protein synthesis, and was also described by Bonilla et al. after a nine week lasting daily intraperitoneal injection of 5 mg Mn/kg b.w. in mice [52]. Although this decrease was observed only in olfactory bulb but not in the striatum, it is still an indication of a disturbed amino acid synthesis due to Mn exposure.

#### Glucose metabolism

Glucose is known to be the main energy supplier for the brain where neurons of the central nervous system can use various intermediates of the glucose metabolism for their maintenance [53]. In the oxidative part of the pentose phosphate pathway (PPP), D-glucose is transformed to ribose-5-phosphate (Fig 4C) via several intermediates, which is used for biosynthesis of nucleotides needed in RNA/DNA synthesis. The observed decrease in ribose-5-phosphate in +Mn samples brings along a lack in nucleotides, which is further aggravating the disturbed RND/DNA synthesis as described above due to the lack of pyrimidines. During the reaction of the oxidative part of the pentose phosphate pathway, NADPH is formed, which keeps up a reducing milieu. Accompanied by a decrease in PPP activity, this reducing milieu might not be maintained any more due to the lack of NADPH formation. These findings are consistent with *in vitro* observations on Mn-treatment of neurons, which failed to compensate for mitochondrial dysfunction by oxidative glucose metabolism shown by an impaired flux of [1-^13^C] glucose through pyruvate dehydrogenase [54].

#### Glutathione metabolism

The disturbance in oxidative defense mechanisms due to Mn exposure becomes also true by the reduction of glutathione (GSH) in brain homogenates compared to control group (Fig 4D). Generation of reactive oxygen species (ROS) and the respective unbalanced anti-oxidative defense has been reported for several neurological disorders. Also during the progression of PD, a lowered GSH level with an increased GSSG/GSH ratio indicates oxidative stress [55]. Although an increase in GSSG was only indicative herein, during a Mn-feeding study in rats we were already able to show this shift towards the oxidized form of GSH [56]. Additionally, we observed a reduction in the GSH precursor molecule γ-glutamylcysteine. This indicates that the synthesis of GSH seems to be disturbed at an early stage and might not solely be based on an inhibition of the enzyme glutathione synthetase. Interestingly, ophthalmic acid was found to be increased in the brain of Mn-treated rats. This GSH analogous peptide has already been discussed to serve as biomarker of oxidative stress [57].

#### Fatty acid synthesis

We also detected four saturated (stearic, behenic, lignoceric, palmitic acid) and six unsaturated fatty acids (oleic, nervonic, dihomo-γ-linolenic, γ-linolenic, docosenoic, docosahexaenoic acid (DHA)) to be decreased in rat brains due to i.v. Mn-injection (Fig 4E). Neuronal membranes are rich in diverse fatty acids where they play an important role for neuronal function maintenance and neurotransmitter signaling. For example, DHA is known to be a potent antioxidant, but in turn, its oxidation products comprise certain toxicity linking its role also to neurodegenerative disorders [58]. Therefore, the observed reduction in these important lipid mediators might be indicative of an enhanced lipid peroxidation in brain tissue due to a Mn-induced milieu of oxidative stress. Other divalent metal ions such as Fe^2+^ or Cu^2+^ were shown to induce hydroxyl radical formation through the Fenton reaction. These hydroxyl radicals can in turn attack polyunsaturated fatty acids (PUFAS) in membrane phospholipids propagating lipid peroxidation [59]. Kapich et al. observed an effective lipid peroxidation of fungal manganese peroxidase on C18 unsaturated fatty acids [60]. This finding is underlining our results, where Mn seemed to evoke lipid peroxidation in the brain due to the reduction in the detected fatty acids. More interestingly, the pool of the major poly-unsaturated fatty acids DHA and arachidonic acid seemed to be depleted while their lipid mediators like PGB1, 15(S)-HETE and ResolvinD2 were found to be increased due to the applied Mn injection. This might mean an activation of inflammatory response via the COX- or LOX- pathway [61], where the latter was found to be involved also in other neurodegenerative diseases such as Alzheimer´s Disease [62].

The discussed alterations in neural metabolisms after the single Mn-injection and their interrelations intend a multifactorial process, which might finally lead to neural inflammation. However, the question regarding cause or effect remains unknown. A previous metabolomic study was carried out by Dorman et al. in Mn-air exposed rhesus monkeys by analysis of blood and urine by means of LC-TOF-MS [39]. The authors reported about 27 metabolites, which were significantly altered in exposed animals, and which were related to oxidative stress and neurotransmission. Some observations were in accordance with our findings such as reduced glutamine, citrullin or adenosylhomocystein. Another recently published work applied an untargeted metabolomics approach in striatal cells investigating the correlation of Huntington Disease (HD) and Mn status [63]. Three different Mn exposure conditions (low, moderate and high/toxic) were investigated for metabolic disturbances by untargeted UPLC-IM-MS in comparison of a wild type and a mutant striatal HD cell line. These HD cells were of certain interest as they have been shown to have decreased ability to accumulate Mn. Interestingly, the authors found decreased pantothenic acid and GSH levels in HD striatal cells, which seemed to be independent from Mn-dependent changes in metabolism. On the other hand, they found a significant change by increased ribulose-5-phosphate for genotype, Mn exposure as well as genotype by Mn exposure interaction effect at the toxic Mn-exposure. It seemed as if the HD cells were hypersensitive to Mn related oxidative stress. The authors conclude that the increase in ribulose-5-phosphate can be associated with an activation of the pentose shunt way leading to production of NADPH, which can in turn increase GSH levels as response against the oxidative stress status observed during HD. In comparison, these defense mechanism might not be activated by the applied concentration of Mn in our study as both, ribose-5-phosphate as well as GSH were found to be significantly lowered in Mn-exposed animals.

Overall, the herein applied ESI-ICR/FT-MS analysis is outstanding with regard to ultrahigh-resolution and very high mass accuracy (<0.1 ppm) [64]. The application of untargeted ESI-ICR/FT-MS analysis therefore facilitated a link between the various altered metabolisms, which was not only successfully applied in studies of neurodegeneration [65, 66] but also for example in context of host-pathogen interactions [67, 68] or other systemic diseases such as obesity [69].

### Correlation studies of findings from the metallomic and the metabolomic approach

**Fig 5:**
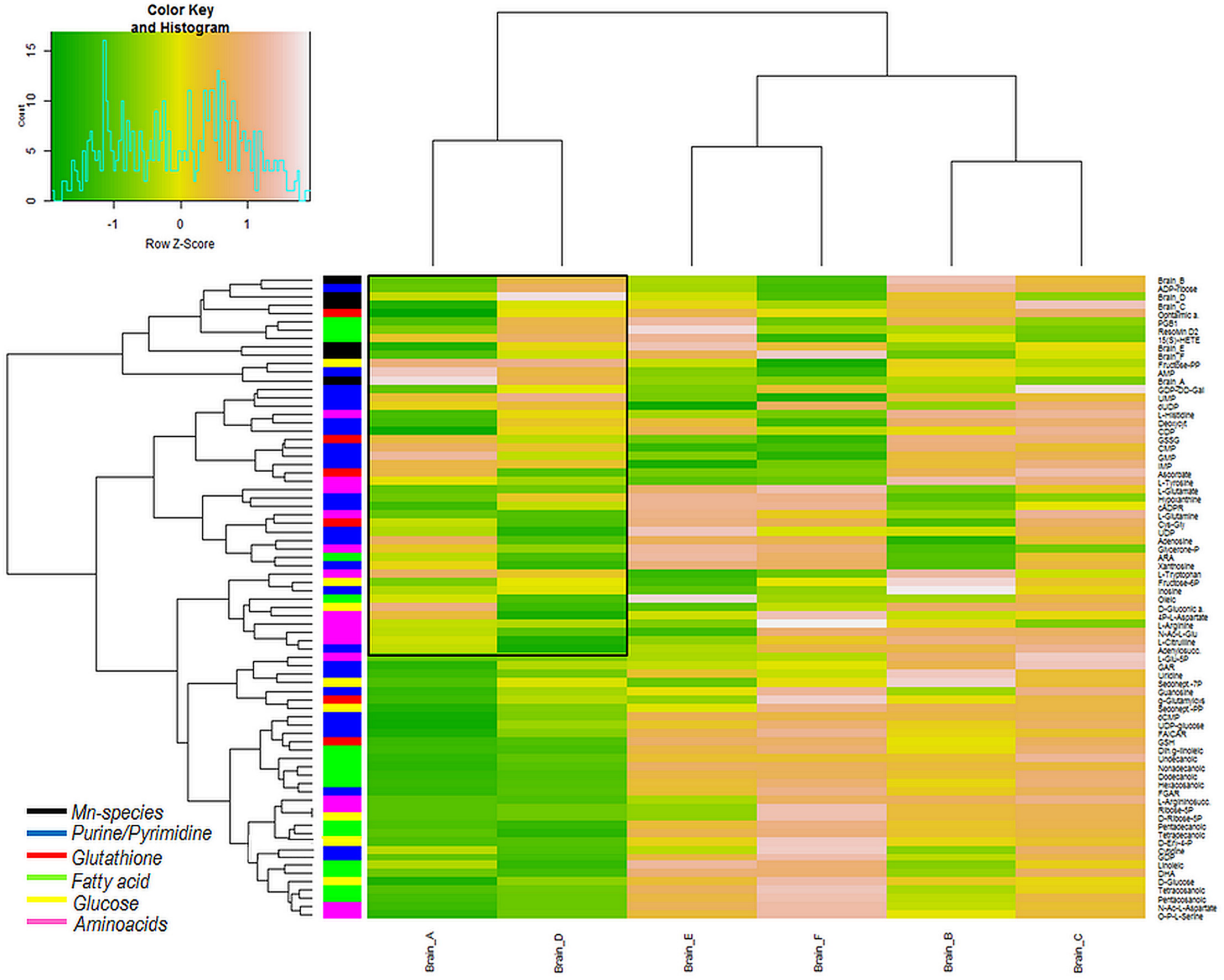
HCA of Mn-species dependent on brain metabolites. HCA was carried out on data from Mn-speciation to observe clustering behavior in relation to changes of brain metabolites (the list of included metabolites can be found in Table B in S2 File). Fractions A and D, E and F as well as B and C were found to cluster together in subgroups.

In order to find eventually occurring correlations between Mn concentrations or Mn-species in brain with detected brain metabolites, we carried out different statistical analyses. In a first step, we applied hierarchical cluster analysis (HCA using Euclidian distance as criteria to cluster the variables) to examine clustering behavior of Mn-species dependent on brain metabolites, which is shown in Fig 5. From the heatmap it can be retrieved that fractions A and D as well as fractions E and F and fractions B and C formed three different subclusters. Although the HMM fraction A clustered together with the Mn-citrate fraction D, the correlations with metabolites differed in few instances in the upper part of the heatmap (enclosed in the black frame). Unless of the co-clustering of fraction A with fraction D, it is interesting that fraction D formed one main cluster compared with the other main cluster formed by the remaining fractions. Overall, in accordance to the total changes of metabolites shown in Fig 4, fraction D was negatively correlated with the majority of the shown metabolites as indicated by the green color. Although fraction A was also found to be negatively correlated with most investigated metabolites, differences could be observed for certain correlations such as for L-Tyrosine, Adenosine or PGB1. The observed positive correlation of fraction D with those biological important metabolites (see discussion above) substantiates the influence of Mn-citrate under conditions of Mn-exposure.

**Fig 6:**
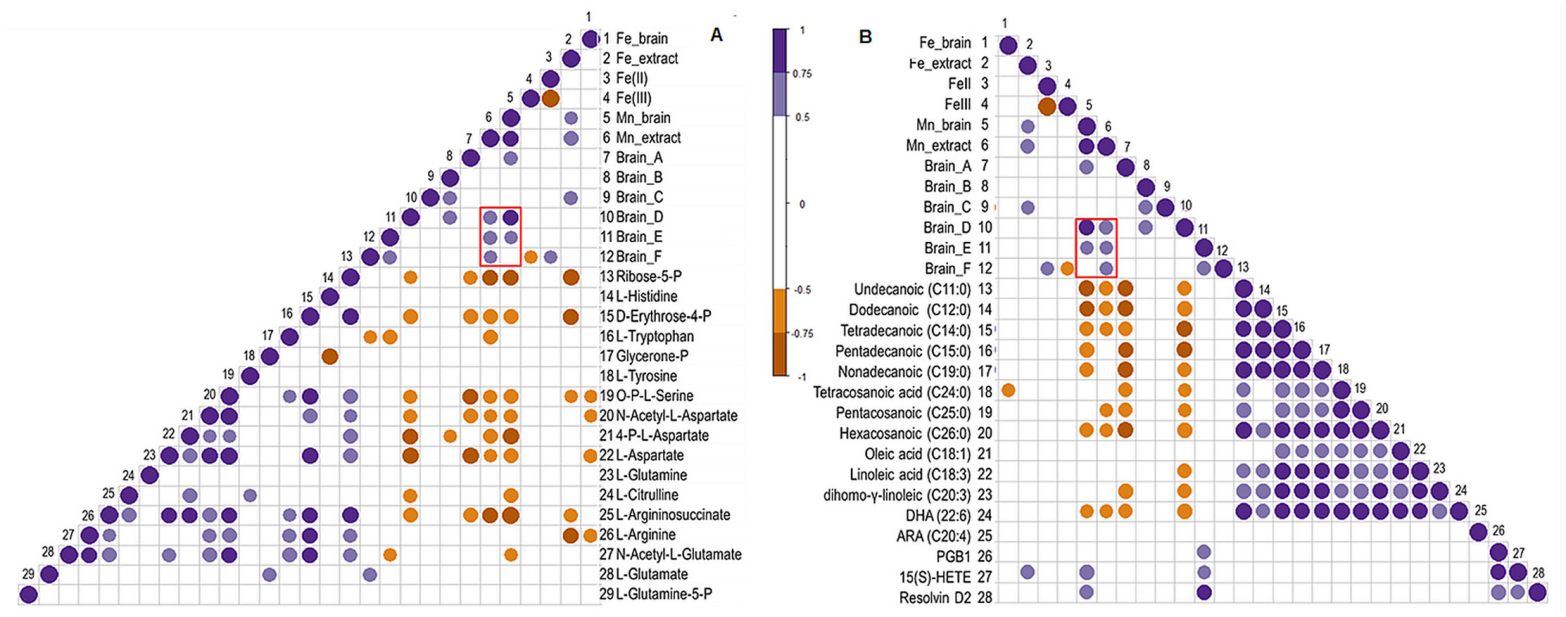
Correlation analysis of different variables from metallomic approach with brain metabolites categorized to brain metabolisms as shown in Fig 4. Pearsons´ correlations were calculated for total Mn, Mn-species, total Fe as well as Fe(II)/(III) with detected brain metabolites for amino acid (A) and fatty acid (B) metabolism. Only the significant correlations are shown, represented as violet (positive correlation) or brown (negative correlation) circles. The different circle size shows the strength of correlation (the bigger the stronger).

In a second step, correlation analyses were carried out based on Pearson Correlations to investigate correlation behavior of Mn-species and other variables with brain metabolites. The correlations were carried out according to the metabolisms shown in Fig 4, whereas only significant correlations are shown by the colored circles (violet positive, brown negative correlation), where the size of the circle gives information about the strength of correlation. In Fig 6A and 6B correlations of variables with metabolites from amino acid and fatty acid synthesis are shown (figures for the correlation analysis of remaining metabolisms can be found in Figs B–D in S2 File).

Additionally shown in the figures is the correlation with total Fe in brain, in brain extracts as well as Fe(II) and Fe(III), which was determined by ion chromatography hyphenated to inductively coupled optical emission spectrometry (IC-ICP-OES) in accordance to the previously published method [56]). Iron homeostasis is of certain interest in Mn-exposure scenarios as altered amounts of Fe or Mn in the brain is resulting in dysregulation or altered homeostasis of the other one (summarized in [70]). As the figures show, herein, Fe in brain extracts was positively correlated with total brain Mn concentrations as well as with Mn in aqueous brain extracts. As the figures also indicate, Fe(II) was inversely correlated with Fe(III), and positively correlated with the inorganic Mn fraction F, although no differences in total concentrations of Fe(II) or (III) were observed between samples from control and Mn-exposed (Fig A in S2 File). In Fig 6, the red frame indicates the positive correlation of total Mn concentrations in brain and brain extracts only with the LMM Mn-species D and E (and inorganic Mn fraction F for Mn in brain extracts), what indicates an increase of these LMM species with increased Mn concentrations in brain. Other correlations were similar to the observation in the HCA as fractions A and D correlated in same manner with the various metabolites: for example negative correlation with amino acids and fatty acids underlining the decrease in these metabolites as shown in Fig 4. This reflects the importance of fraction D (citrate), but also reflects the necessity for future studies in that direction to elucidate the importance of the HMM fraction A as these results were not expected by us. Nevertheless, some other interesting observations could be drawn from this analysis: for example, in Fig 6B, total Mn in brain and brain extracts, as well as fractions A and D correlated negatively with the majority of detected fatty acids. In turn, fraction E (amino acids) showed positive correlation with the breakdown products PGB1, Resolvin D2 and 15(S)-HETE.

Such correlation analysis could help for detection of the influence of certain Mn-species on changes in brain metabolites, which could be indicative of the neural inflammatory status after Mn-exposure. However, as the examples herein show, further research is needed. Such studies need to be carried out in larger sample cohorts as well as in samples, which can be applied in both, speciation as well as metabolomics analysis (e.g. CSF) to have common matrices of observation. In summary, our findings might be a first step towards a new investigation approach of transport procedures of Mn across neural barriers under Mn exposure and the respective connection of these transporters with the alteration in the different neural metabolisms.

## Conclusion

A single low-dose MnCl_2_ i.v. injection in rats was applied to obtain information on both, Mn-species pattern in brain tissue as well as changes in brain metabolisms. Applying SEC-ICP-MS on rat brain extracts, we found a slight shift from HMM compounds towards LMM compounds such as Mn-citrate or –amino acids in samples from Mn-exposed animals. However, the majority of Mn in brain extracts was present as HMM compounds both, in control and Mn-exposed animals. So far, many investigations have been carried out to elucidate neurotoxic effects of Mn. These concentrate on one or few affected mechanisms and mostly contain only knowledge about total Mn concentrations but not about the predominant Mn-species. Herein, measurement of methanolic brain extracts was carried out by means of ultrahigh-resolution ESI-ICR/FT-MS for an untargeted metabolomics analysis of the brain. The power of this analysis is its ability to unravel the various effects of Mn exposure all at once. We were able to observe major changes in various biological pathways in the brain of Mn exposed rats. These included changes in amino acid, glutathione, purin/pyrimidine, and glucose metabolism as well as fatty acid synthesis. It becomes obvious that transition from one altered brain metabolism to another is fluid where changes in one metabolism might be the reason for affecting another one and vice versa. We also correlated Mn concentrations, Mn-species as well as total Fe and Fe(II)/(III) concentrations in brain with detected brain metabolites. That kind of study was a first step towards the investigation of Mn-species, which might have higher or lesser impact on the resulting metabolic changes in brain, what requires extensive further research. The goal would be to determine Mn-carriers, which are more prone for affecting neural metabolism in a biological negative meaning, and use them as early markers of Mn intoxication.

## Materials and Methods

### Chemicals

The list of purchased chemicals include: NH_4_Ac from Merck (Darmstadt, Germany); tris from Carl Roth GmbH+Co.KG (Karlsruhe, Germany); MeOH from Lichrosolv Sigma-Aldrich Chemie (Steinheim, Germany); HNO_3_ (65% p.a., purification by sub-boiling distillation (Berghof, Mühlhausen, Germany)) from Merck (Darmstadt, Germany); human serum albumin (HSA, 99%), bovine γ-globuline, bovine apo-transerrin (98%), manganese(II)chloride tetrahydrate (99.99%), citric acid, reduced and oxidized glutathione, lysozyme, arginase, L-glutamic acid, cysteine from Sigma Aldrich (Steinheim, Germany); multi-elemental standard solution from SCP Science (Quebec, Canada); rhodium single standard from Spex CertiPrep (NJ, USA).

### Animal treatment and sample collection

The animal experiment was in accordance with the institutional Animal Welfare Committee as well as approved by the Bavarian federal state government under the file number 55.2-1-54-2531-180-12. Male Sprague-Dawley rats (RjHan:SD, n = 12) were purchased from Janvier (Janvier S.A.S., France) at the age of three weeks directly after weaning. This ensured sustenance by only mother´s milk. The animals were kept in pairs in polycarbonate cages type III under specified pathogen-free conditions at a 12/12 hours light cycle with paper houses for environmental enrichment receiving a standard diet (ssniff EF R/M AIN93G, Ssniff Spezialdiäten, Soest Germany) and filtered tap water *ad libitum*. After a two week adaption time, animals were anesthetized with isoflurane for a single *i*.*v*. injection: Control rats received 100 μl isotonic saline 0.9% (SteriPharm, Berlin, Germany), while test rats received 100 μl of a solution of 1.5 mg Mn/kg body weight in isotonic saline (sterile filtered) through the tail vein. During a subsequent time of four days, rats were kept in singles while cages were embedded with hemp mats to facilitate quantitative collection of feces for analysis. Four days after the injection, animals were sacrificed by cutting through the *aorta abdominalis* with a ceramic scalpel after deep narcotization with 5% isoflurane, as Mn brain concentrations were found to be highest after four days following injection [71]. At the time point of sacrifice, the animals were aged 39 days (five weeks and four days). Total brains were removed (including olfactory bulb and brain stem, not including spinal cord) and immediately shock frozen in liquid nitrogen until sample preparation. For further sample preparation steps, the whole brain was roughly homogenized under liquid nitrogen, and respective amounts were weighted from this homogenate for brain digestion or extraction. All applied preparation instruments were made of ceramic or PTFE to prevent elemental contamination of tissue.

### Nitric acid digestion of whole brain and measurement by ICP-OES

Procedures for nitric acid digestion of brain tissue and determination of Mn by ICP-OES were analogue to the one described in [56]. In short, 100 mg of coarse homogenated brain tissue (n = 3, under liquid nitrogen) was digested with distilled nitric acid in a Seif apparatus overnight in a heater. The solutions, filled up with aqua bidest, were measured by ICP-OES (Optima 7300 DV, Perkin Elmer, Germany). The analyzed elemental spectral line for Mn was 257.611 nm and for Fe was 259.941 nm with application of a certified multi-elemental standard for quantification. Sample introduction was performed by a peristaltic pump (0.65 mL/min), connected to a seaspray nebulizer fitted into a cyclone spray chamber. Plasma conditions were set to 15 L Ar/min for plasma gas and 0.6 L Ar/min for nebulizer gas at a RF power of 1200W. Blank and standard solutions were measured at the beginning, between control and test samples as well as at the end of the sample series. For quality control, certified reference material (bovine liver, BCR 185; n = 3) was digested and measured in a row with the samples. The recovery obtained for Mn was 94.7±0.9% and for Fe was 94.9±0.6% compared with declared values (9.3±0.3 μg/g and 214±5 μg/g, respectively).

#### Extraction for Mn-speciation by SEC-ICP-MS

The extraction of brain tissue was carried out analogous to the one developed by Diederich et al. for specific extraction of Mn [24]. 0.5–1 g of coarse homogenated brain (n = 2 per brain) was homogenized with 4 mL of ice cold extraction buffer (10 mM Tris-HCl, pH 7.4, purged with helium for 3h) in a 10 mL glass homogenizer (Fortuna, Neolab, Germany) under Ar atmosphere (Atmos Bag, Sigma-Aldrich). After centrifugation (5 minutes at 1434*xg* at RT and 45 minutes at 20160*xg* at 2°C, Biofuge 17 RS, Heraeus-Sepatech, Osterode), the supernatant was transferred into an Eppendorf tube while the pellet was resuspended in extraction buffer and centrifuged again (30 minutes at 20160*xg* at 2°C). The supernatants were pooled and frozen at -80°C until analysis while the pellets were kept at 4°C until microwave digestion. Pellets were digested with 5 mL distilled nitric acid by a special microwave program (0–5 W for 5 minutes, 500 W for 10 minutes, 500–1000 W for 5 minutes, 1000 W for 45 minutes and back to 0 W within 15 minutes; Microwave3000, Anton Paar, Austria). Filled up samples (50 mL, aqua bidest) were stored at RT until analysis.

#### Extraction for ESI-ICR/FT-MS measurement

For ESI-ICR/FT-MS measurement methanolic brain extraction was carried out as described in [56]. In short, after 20 minutes sonication on ice of 50 mg coarse homogenized brain in 500 μL 50% MeOH, samples were homogenized in a 2 mL glass homogenizer (Fortuna, Neolab, Germany) with addition of 250 μL 50% MeOH. Subsequently, homogenates were transferred back into the Eppendorf tube with 250 μl of 50% MeOH and sonicated again on ice for 20 minutes. After centrifugation for 30 minutes at 2°C and 18900*xg*, the supernatant was diluted with 70% MeOH (1:10) and extracts were stored at -80°C until measurement.

#### ICP-sf-MS analysis of brain extracts and pellets

For determination of elemental concentrations of Mn brain extracts and pellets ICP-sf-MS analysis was carried out due to low elemental concentrations of Mn (Element 2, Thermo Fisher Scientific, Germany). Brain extracts were thawed at 4°C overnight and diluted 1:10 with aqua bidest while the nitric acid digested solutions of pellets were measured undiluted, both with 1 μg/L Rh as internal standard. Medium resolution mode (R = 4000) was applied with detection of ^55^Mn and ^103^Rh. Plasma conditions were 15 L Ar/min for plasma gas, 1.45 L Ar/min for auxiliary gas and 1.14 L Ar/min for sample gas at a RF power of 1260 W. Before measurement, a so called peak search with a 1 μg/L Rh solution was carried out to set the respective mass offset for each isotope for correct evaluation of masses. A five point calibration (0, 100, 250, 500 and 1000 μg/L containing 1 μg/L Rh as internal standard) with a multi elemental standard containing Mn was carried out and the calibration line with at least r^2^ = 0.999x was used for calculation of sample concentrations.

Comparability between ICP-sf-MS and ICP-OES was checked by analyzing certified reference material (bovine liver, BCR 185; n = 3) on both instruments after microwave digestion. Measured levels for Mn on both instruments were exactly the same with a recovery of 94.7±0.9% compared with declared values.

#### ESI-ICR/FT-MS measurement of brain extracts

Changes in brain metabolites were determined by means of electrospray ionization fourier transform ion cyclotron resonance mass spectrometry (ESI-ICR/FT-MS, Solarix, Bruker, Bremen, Germany; electrospray source: ESI, Apollo II; Bruker Daltonics, Bremen, Germany) equipped with a 12-T superconducting magnet (Magnex Scientific, Varian Inc., Oxford, UK). Measurements were carried out in negative ionization mode in a mass to charge ration of 123–1000 with an ion accumulation time of 300 ms. The liquid flow rate was 2 μl/min at a temperature of 180°C with an ESI voltage difference of 3500 V between electrode and counter electrode and an additional voltage drop of 500 V between counter electrode and inner cone for further acceleration of ions. The dry gas flow rate was maintained at 4 L/min and the ESI nebulizer gas flow rate was kept at 2 L/min. External mass calibration was performed by analyzing an 3 mg/L arginine solution in MeOH with calibration errors below 0.1 ppm (analyzed arginine clusters: [M-H]- m/z 173.10440, 347.21607, 521.32775, 695.43943).

The ICR-FT-MS raw spectra were processed with DataAnalysis Version 4.1 (Bruker Daltonik GmbH, Bremen, Germany). First, they were calibrated internally with an error below 0.1 ppm by using a list of lipids with known exact masses. After calibration, spectra were exported to peak lists at a signal to noise ratio (S/N) of 4 and an intensity threshold of 0.01%. The mass spectra were aligned through an in-house written software within an error of 1 ppm. The resulting data matrix was uploaded to MassTRIX web server [30] in order to perform annotation of masses within an error range of one ppm. Further statistical data treatment is described below.

#### SEC-ICP-MS of brain extracts

For size exclusion chromatography of Mn-species in brain extracts, a Knauer 1100 Smartline inert Series gradient HPLC system was used with two serially installed SEC columns: Biobasic 300mesh column (300x8mm ID, Thermo Fisher Scientific, Germany; separation range 700–5 kDa) and a 250x8 mm ID peak column filled with Toyopearl HW40S (TosoHaas, Stuttgart, Germany) (separation range 100–2000 Da). This column combination provided separation of Mn-proteins from each other and from Mn-citrate as well as the latter from another LMM compound and from inorganic Mn. A two-phase eluent consistent of 90% A (50 mM NH_4_Ac, pH 5.6) and 10% B (10mM Tris, 500mM NH_4_Ac, 5% MeOH, pH 8) was applied at an isocratic flow rate of 0.7 ml/min and the injection volume of the samples was 25 μl. The column effluent passed an UV detector before introduction into a Meinhard nebulizer fitted in a laboratory made spray chamber at the ICP-MS (NexIon300, Perkin Elmer, Rodgau-Jügesheim, Germany) for detection of ^55^Mn. The RF power was set to 1250 W, the plasma gas was 15 L Ar /min while nebulizer gas was optimal at 0.94 L Ar/min and dwell time was 350 ms. The system was run in DRC (dynamic reaction cell) mode with NH_3_ as DRC gas at 0.58 ml/min and the DRC band pass (q) at 0.45.

For column mass calibration, standard solutions were injected consisting of: alpha-2-macroglobulin (725 kDa), ferritin (440 kDa), γ-globulin (190 kDa), arginase (107 kDa), transferrin (78 kDa), human serum albumin (66 kDa), glutamine synthetase (42 kDa), oxidized glutathione (612 Da), citrate (192 Da), and inorganic Mn (55 Da). By comparison of UV and ICP-MS signal, the respective retention times were recorded. The two obtained calibration equations were ln(MW) = -0.577xRT+14.551 (r^2^ = 0.999) for compounds eluting before 20 minutes and ln(MW) = -0.4339xRT+8.4392 (r^2^ = 0.994) for compounds eluting after 20 minutes. The void volume was seven minutes as determined by application of blue dextran (2000 kDa). Mn-standard solutions were prepared from 1000 mg/L stock solutions of protein (HSA, γ-globulin, transferrin, ferritin and citric acid) mixed (4+1) with 5 g/L Mn from MnCl_2_xH_2_O in eluent A. After complexation for one week at room temperature, aliquots were stored at -20°C in the dark and working solutions were prepared freshly.

Samples were thawed at 4°C overnight before analysis and applied undiluted. After every third run, columns were cleaned separately in reversed flow by a solution of 20% 500 mM NaCl and 80% MeOH at 0.1 ml/min to remove elemental as well as organic residues. Obtained sample chromatograms were elaborated with PeakFit^TM^ v4.11 (Systat, Erkrath, Germany) by calculations of peak area. Column recovery was determined by spiking of brain extracts with different concentrations of a Mn single standard and was found to be 82–128%. LoD was calculated by the 3σ-criterion and was 17 ng/L.

#### Statistical evaluation

Significance between Mn-species in control and Mn-injected brain extracts were calculated by student´s t-test in Microsoft excel after application of Grubbs test to determine outliers. A two-way ANOVA including the interaction effect between group (i.e. control or +Mn_Inj) and SEC-fractions (i.e. A-F) was carried out to elucidate significant changes in SEC-fractions. P-values ≤0.05 were considered to be statistically significant (*p≤0.05, **p≤0.01, ***p≤0.001). The ESI-ICR/FT-MS data were analyzed with different multivariate techniques in order to extrapolate and visualize significant features, related to the different nature of the experimental groups. Orthogonal partial least square discriminant analysis (OPLS-DA) was done on scaled data (Unit Variance). The elaborations were done in SIMCA-P 13.0.3.0 (Umetrics, Umea, Sweden) and the HCA heatmap or correlation figures with the R Package 3.1.1.

## Supporting Information

S1 File **Fig A**. Analysis of feces.; **Text A**. Short description for analysis of feces.
(DOCX)

S2 File **Table A**. p-values according to the two-way ANOVA of results from Mn-speciation; **Table B**. List with metabolites according to the appearance in the heatmap from HCA in Fig 5; **Fig A**. Concentrations of Fe in brain, brain extracts and pellets (left) as well as of Fe(II) and Fe(III) in brain extracts determined by IC-ICP-OES (right); **Fig B**. Correlation analysis of results from metallomics with glucose metabolism; **Fig C**. Correlation analysis of results from metallomics with glutathione metabolism; **Fig D**. Correlation analysis of results from metallomics with purine/pyrimidine metabolism.
(DOCX)

## Abbreviations:

PD: Parkinson’s Disease
LMM, HMM: Low/High molecular mass
SEC-ICP-MS: Size exclusion chromatography-inductively coupled plasma-mass spectrometry
ESI-ICR/FT-MS: electrospray ionization—ion cyclotron resonance-Fourier transform-mass spectrometry
CSF: Cerebrospinal fluid
