# Supplementary material for: Changes in Brain Metallome/Metabolome Pattern due to a Single i.v. Injection of Manganese in Rats
Source: PLoS One. 2015 Sep 18;10(9):e0138270. doi: 10.1371/journal.pone.0138270 (PMC4575095; doi:10.1371/journal.pone.0138270)
Supplement: S1 File — Fig A. Analysis of feces.; Text A. Short description for analysis of feces. (DOCX) [file pone.0138270.s001.docx]

S1 File


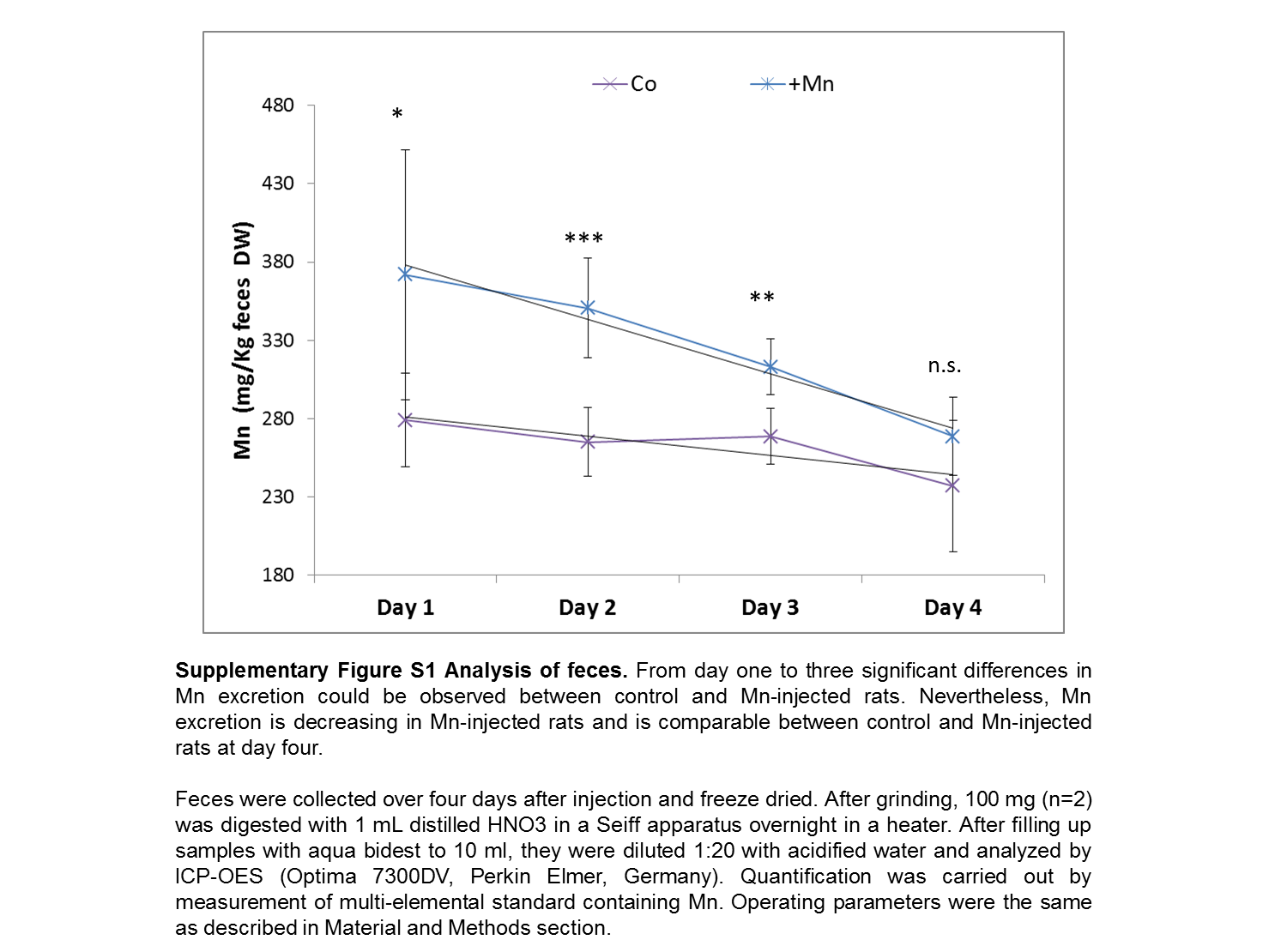


***S1 Fig A. Analysis of feces.*** *From day one to three significant differences in Mn excretion could be observed between control and Mn-injected rats. Nevertheless, Mn excretion is decreasing in Mn-injected rats and is comparable between control and Mn-injected rats at day four.*

***S1 Text. Short description for analysis of feces.*** *Feces were collected over four days after injection and freeze dried. After grinding, 100 mg (n=2) was digested with 1 mL distilled HNO3 in a Seiff apparatus overnight in a heater. After filling up samples with aqua bidest to 10 ml, they were diluted 1:20 with acidified water and analyzed by ICP-OES (Optima 7300DV, Perkin Elmer, Germany). Quantification was carried out by measurement of multi-elemental standard containing Mn. Operating parameters were the same as described in Material and Methods section.*
