# Supplementary material for: Changes in Brain Metallome/Metabolome Pattern due to a Single i.v. Injection of Manganese in Rats
Source: PLoS One. 2015 Sep 18;10(9):e0138270. doi: 10.1371/journal.pone.0138270 (PMC4575095; doi:10.1371/journal.pone.0138270)
Supplement: S2 File — Table A. p-values according to the two-way ANOVA of results from Mn-speciation; Table B. List with metabolites according to the appearance in the heatmap from HCA in Fig 5; Fig A. Concentrations of Fe in brain, brain extracts and pellets (left) as well as of Fe(II) and Fe(III) in brain extracts determined by IC-ICP-OES (right); Fig B. Correlation analysis of results from metallomics with glucose metabolism; Fig C. Correlation analysis of results from metallomics with glutathione metabolism; Fig D. Correlation analysis of results from metallomics with purine/pyrimidine metabolism. (DOCX) [file pone.0138270.s002.docx]

S2 File


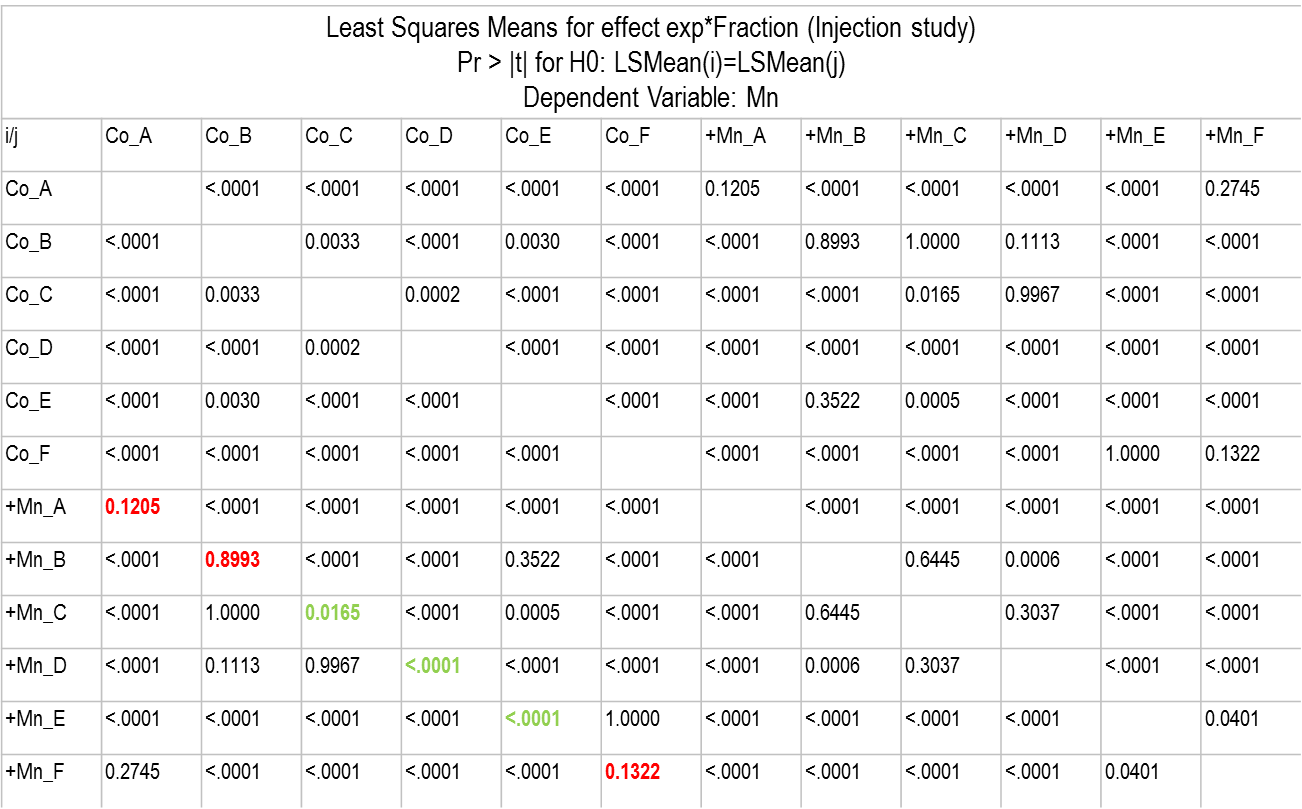


S2 Table A. P-values for two-way ANOVA of Mn-speciation results. The model also included the interaction effect between group (i.e. control or Mn-exposed) and SEC fraction. The only significant results were obtained for fractions C, D and E, which is in line with results from fold-changes in comparison of control and exposed by t-test.

| Brain_B | Adenylosuccinate |
| --- | --- |
| ADP-ribose | L-Glutamine-5-Phosphate |
| Brain_D | GAR (glycinamide ribotide) |
| Brain_C | Uridine |
| Ophthalmic acid | Sedoheptulose-7-Phosphate |
| PGB1 | Guanosine |
| Resolvin D2 | gamma-Glutamylcysteine |
| 15(S)-HETE | Sedoheptulose-diphosphate |
| Brain_E | dCMP |
| Brain_F | UDP-glucose |
| Fructose-diphosphate | FAICAR (5-Formamidoimidazole-4-carboxamide ribotide) |
| AMP | GSH (Glutathione) |
| Brain_A | Dihydro-gamma-linoleic acid |
| GDP-didesoxy-Galactose | Undecanoic acid |
| UMP | Nonadecanoic acid |
| dUDP | Dodecanoic acid |
| L-Histidine | Hexacosanoic acid |
| Deoxycytidine | FGAR (formylglycinamide ribotide) |
| CDP | L-Argininosuccinate |
| GSSG | Ribose-5-Phosphate |
| CMP | D-Ribose-5-Phosphate |
| IMP | Pentadecanoic acid |
| Ascorbate | Tetradecanoic acid |
| L-Tyrosine | D-Erythrose-4-Phosphate |
| L-Glutamate | Cytidine |
| Hypoxanthine | GDP |
| cADP-Ribose | Linoleic acid |
| L-Glutamine | DHA (docosahexanoic acid) |
| Cysteinyl-Glycin | D-Glucose |
| UDP | Tetracosanoic acid |
| Adenosine | Pentacosanoic acid |
| Glycerone-Phosphate | N-Acetyl-L-Aspartate |
| Arachidonic acid (ARA) | O-Phospho-L-Serine |
| Xanthosine |  |
| L-Tryptophan |  |
| Fructose-6-Phosphate |  |
| Inosine |  |
| Oleic acid |  |
| D-Gluconic acid |  |
| 4-Phospho-L-Aspartate |  |
| L-Arginine |  |
| N-Acetyl-L-Glutamate |  |
| L-Citrulline |  |

S2 Table B. Metabolites in the order of appearance in the heatmap from HCA in Fig 5.


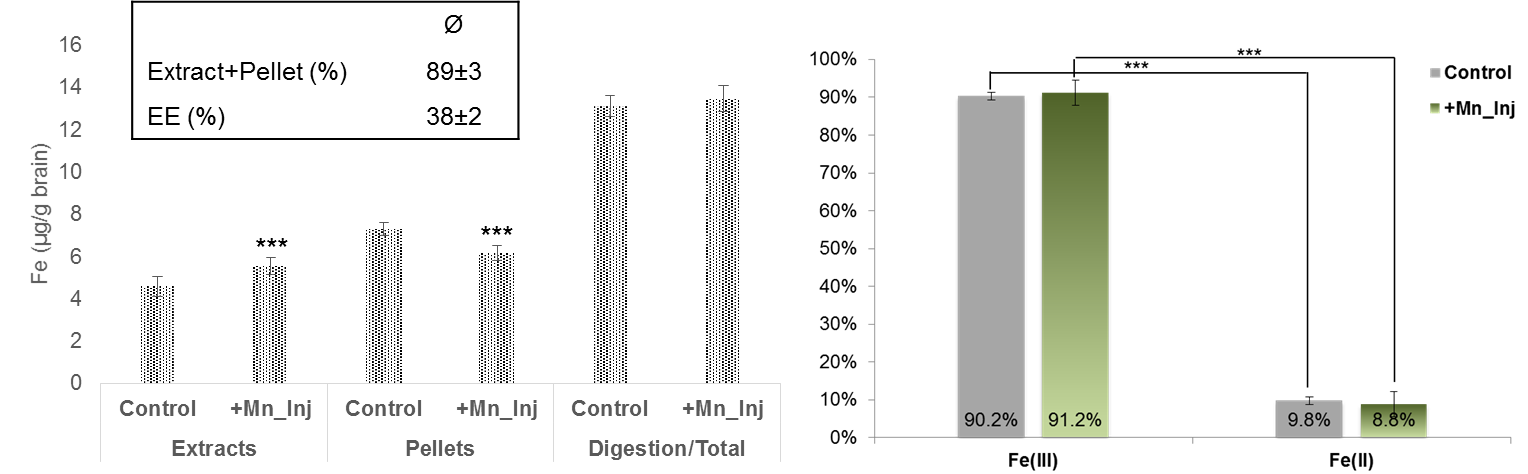


S2 Fig A. (Left) Determination of Fe in aqueous extracts, pellets and total brain (also showing the recovery and extraction efficiency (EE) in the black framed box. Fe was significantly increased in brain extracts and significantly decreased in pellets from extraction, which revealed no difference in Fe in total brain between control and +Mn-Inj samples. (Right) Fe(II) and Fe(III) was determined by IC-ICP-OES in brain extracts as described in [56] and revealed no difference in concentrations between control and Mn-exposed animals.


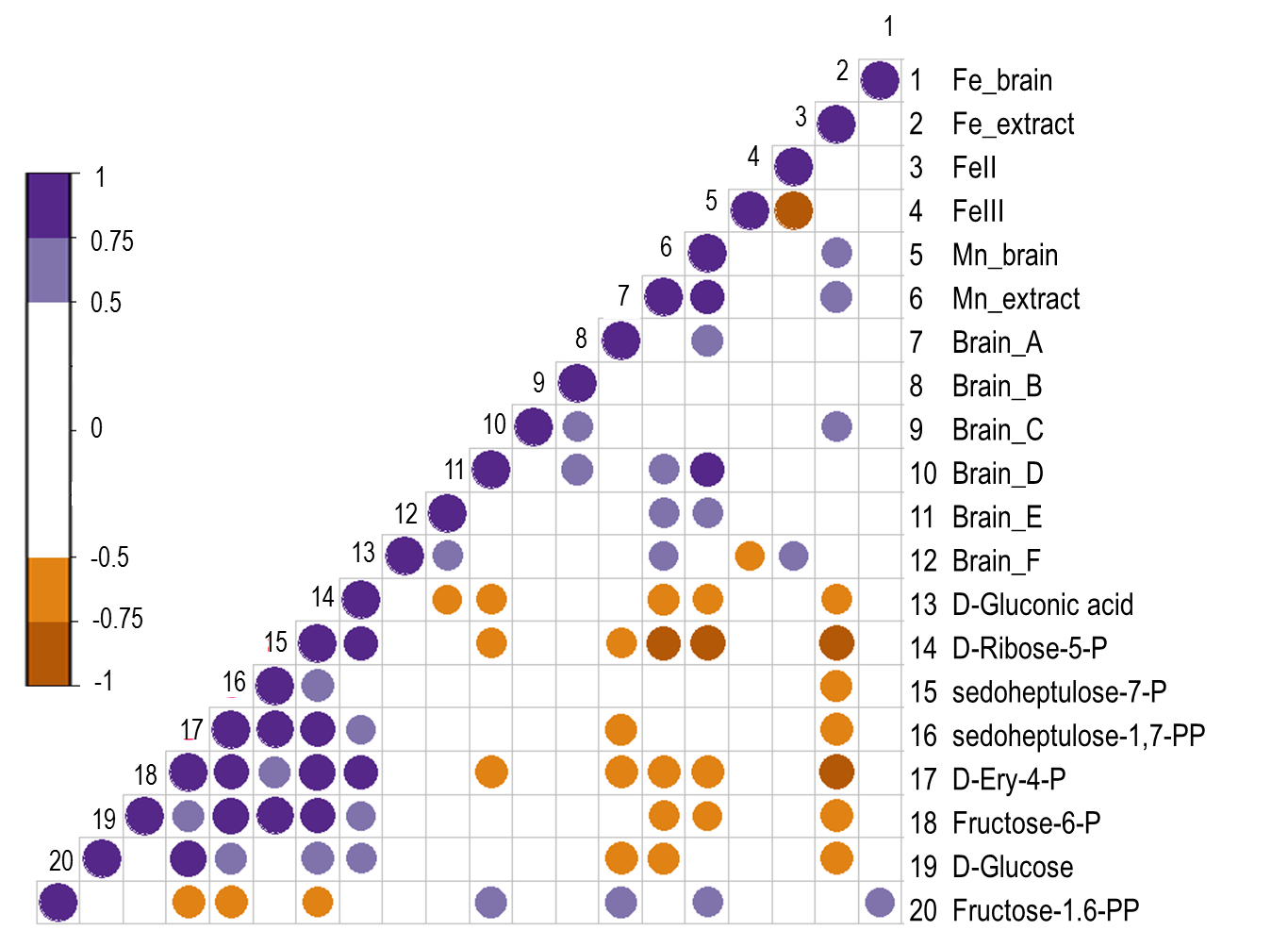


S2 Fig B. Correlation analysis of variables from the metallomic approach with metabolites from glucose metabolism. Interestingly, Fe in extract is negatively correlated with all detected metabolites except for Fructose-1.6-bisphsphate. For example, Fe(II) is positively correlated with Mn in brain and brain extracts.


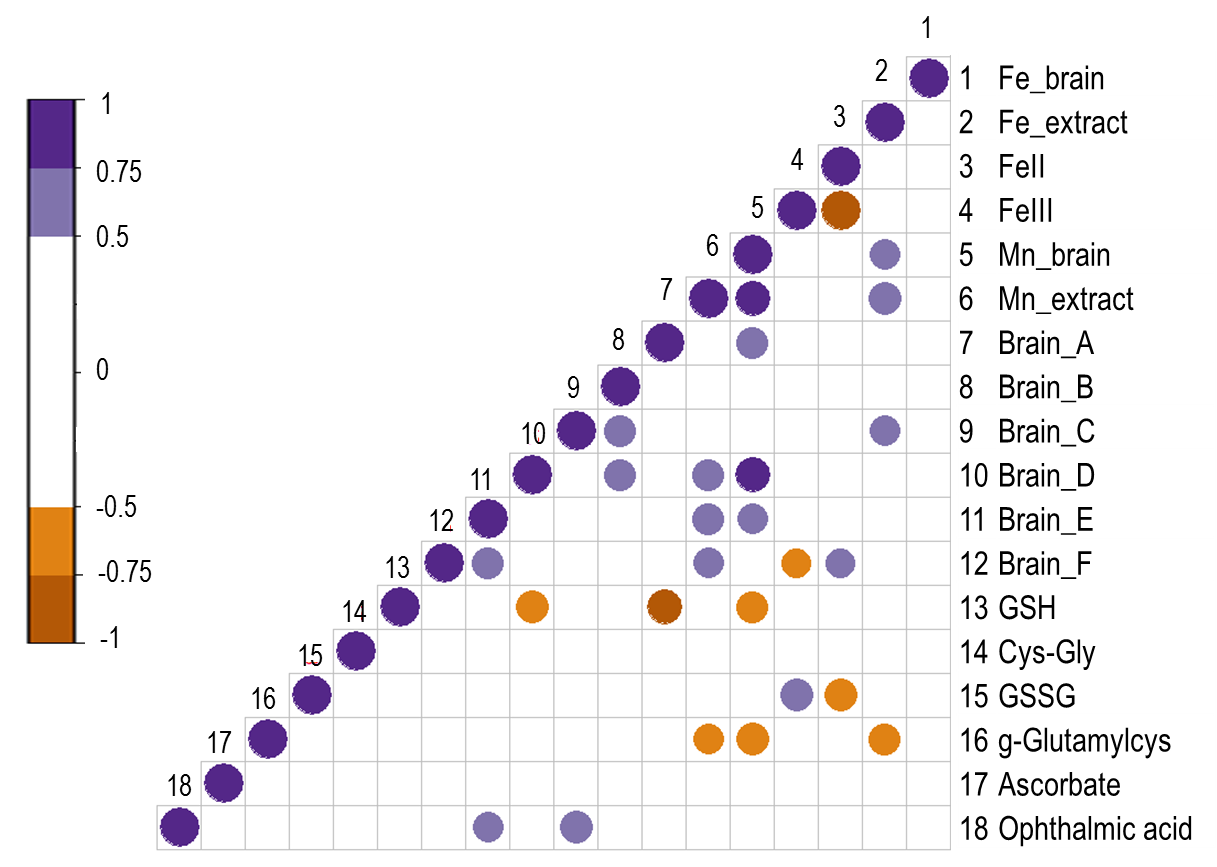


S2 Fig C. Correlation analysis of variables from the metallomic approach with metabolites from glutathione metabolism. Mn in total brain, SEC fractions A and D are negatively correlated with GSH.


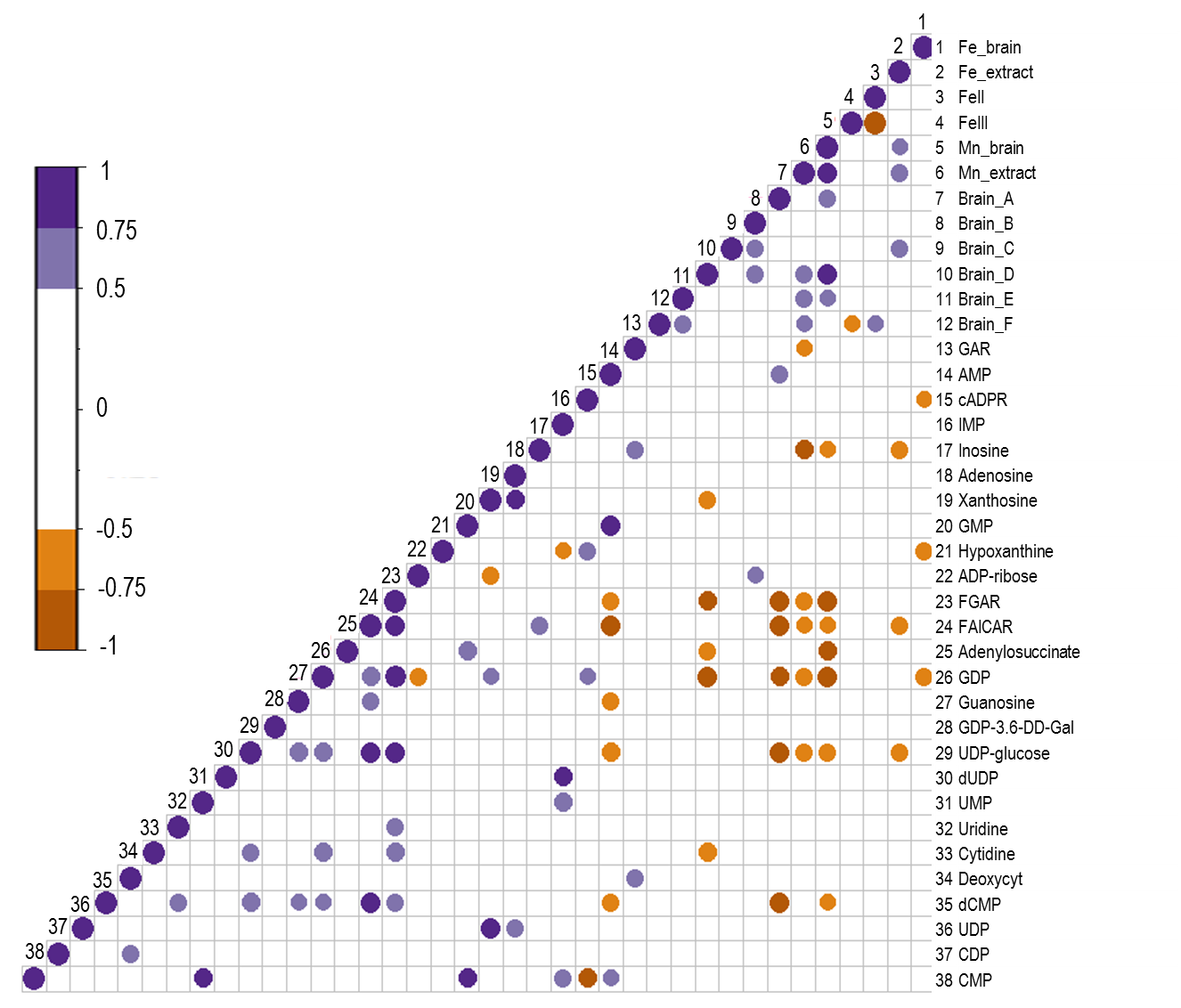


S2 Fig D. Correlation analysis of variables from the metallomic approach with metabolites from purine and pyrimidine metabolism.
